# Cancer Treatment by Caryophyllaceae-Type Cyclopeptides

**DOI:** 10.3389/fendo.2020.600856

**Published:** 2021-01-14

**Authors:** Mohammad Hassan Houshdar Tehrani, Mohammadreza Gholibeikian, Abdolhamid Bamoniri, Bi Bi Fatemeh Mirjalili

**Affiliations:** ^1^ Department of Pharmaceutical Chemistry, School of Pharmacy, Shahid Beheshti University of Medical Sciences, Tehran, Iran; ^2^ Department of Organic Chemistry, Faculty of Chemistry, University of Kashan, Kashan, Iran; ^3^ Department of Chemistry, College of Sciences, Yazd University, Yazd, Iran

**Keywords:** anticancer activity, Caryophyllaceae-type cyclopeptides, dianthins, longicalycinin A, plant family, synthetic analogues

## Abstract

Cancer is one of the leading diseases, which, in the most cases, ends with death and, thus, continues to be a major concern in human beings worldwide. The conventional anticancer agents used in the clinic often face resistance among many cancer diseases. Moreover, heavy financial costs preclude patients from continuing treatment. Bioactive peptides, active in several diverse areas against man’s health problems, such as infection, pain, hypertension, and so on, show the potential to be effective in cancer treatment and may offer promise as better candidates for combating cancer. Cyclopeptides, of natural or synthetic origin, have several advantages over other drug molecules with low toxicity and low immunogenicity, and they are easily amenable to several changes in their sequences. Given their many demanded homologues, they have created new hope of discovering better compounds with desired properties in the field of challenging cancer diseases. Caryophyllaceae-type cyclopeptides show several biological activities, including cancer cytotoxicity. These cyclopeptides have been discovered in several plant families but mainly are from the Caryophyllaceae family. In this review, a summary of biological activities found for these cyclopeptides is given; the focus is on the anticancer findings of these peptides. Among these cyclopeptides, information about Dianthins (including Longicalycinin A), isolated from different species of Caryophyllaceae, as well as their synthetic analogues is detailed. Finally, by comparing their structures and cytotoxic activities, finding the common figures of these kinds of cyclopeptides as well as their possible future place in the clinic for cancer treatment is put forward.

## Introduction

Cancer cells, often present as malignant tumors, are described as compiled abnormal cells with fast growth and division in an uncontrolled manner (1). They may migrate and invade every part of the body, a phenomenon that is named metastasis (1). Cancer cells do not die, and so apoptosis, a programmed cell death that occurs in normal cells, does not happen in cancerous cells, and as a result, any old, damaged, and defective cells survive and come together with newly born unwanted cells (2). The progression of cancer and its metastasis ultimately ends with patient death. On the basis of a WHO report, it is estimated that 1 in 6 deaths globally is due to cancer (3). As time passes, the incidence of cancer cases increases year by year such that it will involve 29.5 million people in 2040 (4).

Cancer treatment by traditional chemotherapeutics may have several drawbacks, including the danger of disease recurrence due to emerging resistance to these agents and the appearance of drug side effects during long-term usage (1). Therefore, cancer treatment requres changing of anticancer drug protocols many times, which obviously imposes high costs and a major economic burden on the patients and their relatives (5). The problem is compounded if there are social and emotional pressures on the patients (5).

The successful use of peptides for treating various diseases (6, 7) has attracted researchers to apply these agents to combating cancer (8, 9). That is because peptides have several advantages over other chemotherapeutics. These noticeable characteristics of peptides may be enumerated as good efficacy, high potency, and low immunogenicity (1). Peptides are amenable to many changes in their sequences in a way of showing selective action in targeted cells while causing low toxicity to normal cells (1). In addition, peptides cause low incidence of resistance in malignant cells (10). However, peptides contain some properties that are considered to be disadvantages (1, 9). Among these unfavorable characteristics, low intolerability against lytic enzymes is the most important feature (1). To improve low stability, some strategies have been suggested by researchers (11). These are reordering peptide sequences (12), choosing D-amino acid instead of L-amino acid (13, 14), and converting linear peptide into a cyclized form (15). Cyclization can also cause peptide conformation to become more suitable for binding to a peptide biologically active site (16).

Cyclic peptides (also called cyclopeptides) have many potential therapeutic properties (17) and are suitable to be used as drugs in the clinic (18). The plant cyclopeptides show many attractive biological activities. They are divided into eight types (19). Among them, Caryophyllaceae-type cyclopeptides composed of cyclo di-, penta-, hexa-, hepta-, octa-, nona-, deca-, undeca-, and dodeca-amino acid residues are mounted by more than 200 kinds (20). These cyclopeptides are extracted from several plant families, mainly from Caryophyllaceae. This is a large family of flowering plants that contains approximately 81 genera and 2625 species (21). Discovery of cyclopeptides from Caryophyllaceae dates back to 1959, when the first cyclopeptide, named Cyclolinopeptide A, was extracted from the seeds of *Linum usitatissimum* (22). It is a potent immunosuppressive agent (23). Since then, Caryophyllaceae-type cyclopeptides, defined as homomonocyclopeptides, have been discovered from higher plants. These peptides show several biological activities, such as antimalarial, antiplatelet, immunomodulating, immunosuppressive, cyclooxygenase inhibitory, tyrosinase and melanogenesis inhibitory, Ca^2+^ antagonism, and estrogen-like and cytotoxic activities (19, 24). Demonstrating various activities, including an anticancer effect, by Caryophyllaceae-type cyclopeptides encouraged us to review and summarize the latest biological findings on these peptides. In this review, the focus is on discussing the cytotoxic action of Caryophyllaceae-type cyclopeptides, especially dianthins and their synthetic analogues. Moreover by this review, attempt will be made to obtain information about the relations between structure and anticancer activity (SAR) of the cyclic peptides, which is useful for finding good candidates among cyclopeptides as anticancer agents with optimum structures for application in the clinic.

## Segetalins

**Chart 1:**
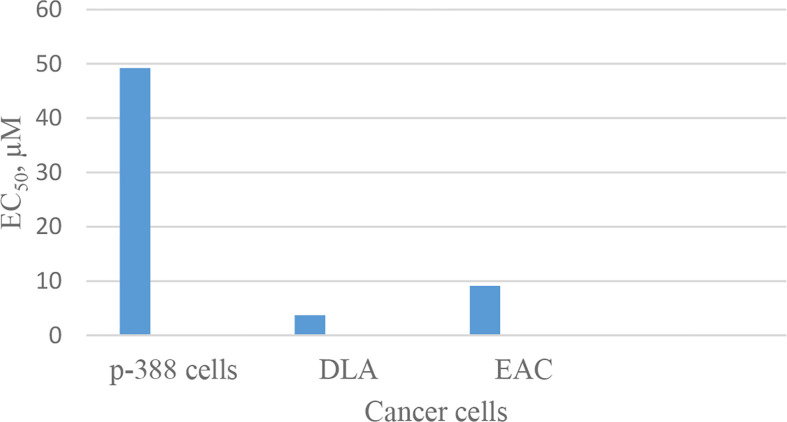
Inhibitory action of segetalin E against cancer cells.

**Figure 1:**
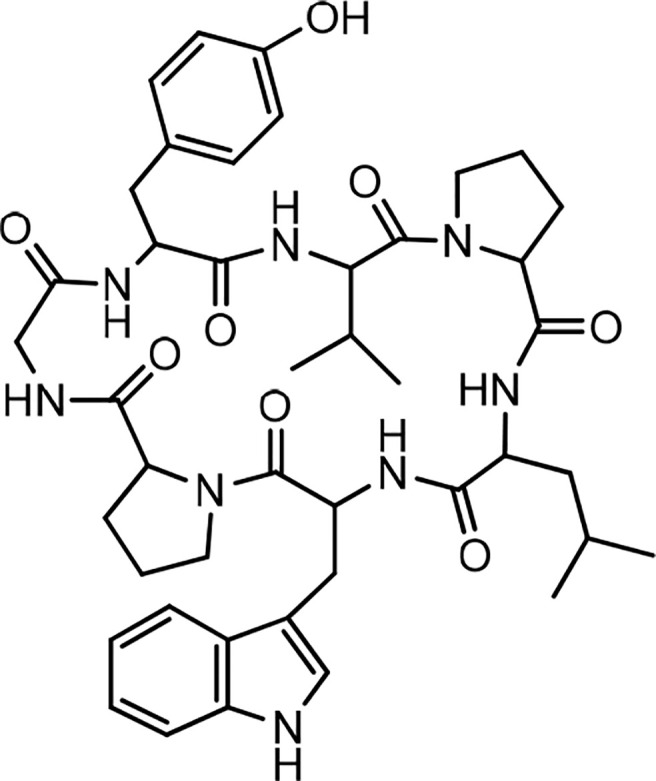
Structure of segetalin E.

Discovery of segetalin cyclopeptides from the seeds of *Vaccaria segetalis* (Caryophyllaceae) started in 1994, when the first cyclopeptide, called segetalin A, was isolated, and its structure was proved by instrumental analyses, i.e., two-dimensional nuclear magnetic resonance (2D NMR) and electrospray ionization mass spectrometry (ESI-MS)/MS as well as by chemical and enzymatic hydrolysis methods. The sequence of its structure was found to be cyclo(Ala-Gly-Val-Pro-Val-Trp-). It was shown that this cyclopeptide produces an estrogenic activity on the uterine weight of ovariectomized rats (25). Soon after, the isolation of other segetalins, such as segetalins B, C, and D, was reported from the same plant (26). The estrogenic activity of segetalin B was higher than segetalin A, whereas segetalins C and D did not show such activity noticeably (26). The resemblance of the part of the peptide sequence between segetalin A and segetalin B (Gly-Val and Trp-Ala) was suggested to be the reason for such similar activity. (The peptide sequences are given in **Table 1**.) Discovery of the natural segetalins E, F, G, and H from *Vaccaria segetalis* is documented in several reports (27–30). Further examining the biological activity, segetalins G and H in addition to A and B displayed estrogenic activity in ovariectomized rats (27, 31). It was assumed that this property was due to the kind of sequence as well as conformation of the peptides (31). The sequences of Trp-Ala-Gly-Val or Tyr-Ala-Gly-Val, β-turn occurring, one in segetalin B (between Trp^4^ and Ala^5^) and two in segetalin A (between Trp^5^ and Ala^6^ and between Val^2^ and Pro^3^) as well as cyclic rather than acyclic shapes of the peptides are important factors in demonstrating estrogenic activity (31). When the biological activity of segetalin E (**Figure 1**) was examined on cancer cells using an MTT (3-(4,5-dimethylthiazol-2-yl)-2,5-diphenyltetrazolium bromide) assay, it was found that segetalin E had moderate inhibitory activity against the growth of lymphocytic leukemia P-388 cells (*IC_50_* 40 µg/mL^-1^ ≈ 49.2 µM) (32) and higher inhibitory actions against Dalton’s lymphoma ascites (DLA) and Ehrlich’s ascites carcinoma (EAC) cell lines (with *IC_50_* values of 3.71 and 9.11 µM, respectively) (29) (**Chart 1**).

**Table 1:** Segetalin cyclopeptides with their sequences and biological activities.

Peptide names	Peptide sequence	Cytotoxic activity	Pharmacological activities	Anti-infective activities
Segetalin A	Cyclo(-Gly-Val-Pro-Val-Trp-Ala-)	—	Estrogenic	
Segetalin B	Cyclo(-Gly-Val-Ala-Trp-Ala-)	—	Estrogenic, Contractile	
Segetalin C	Cyclo(-Gly-Leu-His-Phe-Ala-Phe-Pro-)	—		
Segetalin D	Cyclo(-Gly-Leu-Ser-Phe-Ala-Phe-Pro-)	—		
Segetalin E	Cyclo(-Gly-Tyr-Val-Pro-Leu-Trp-Pro-)	On P-388 cells, DLA cells, EAC cells		Antihelimintic
Segetalin F	Cyclo(-Tyr-Ser-Ser-Lys-Pro-Ser-Ala-Ser)		Vasorelaxant	
Segetalin G	Cyclo(-Gly-Val-Lys-Tyr-Ala)		Estrogenic, Vasorelaxant	
Segetalin H	Cyclo(-Gly-Tyr-Arg-Phe-Ser)		Estrogenic, Vasorelaxant	
Segetalin J	Cyclo(-Phe-Gly-Thr-His-Gly-Leu-Pro-Ala-Pro-)	—		
Segetalin K	Cyclo(-Gly-Arg-Val-Lys-Ala-)	—		

Segetalin E also showed antihelmintic activity against two earthworms *M. konkanensis* and *P. corethruses* by a dose of 2 mg/mL. In another study, the structure as well as absolute stereochemistry of segetalin F were determined by analytical and chemical means (30). In an experiment designed to determine the vasorelaxant activity of segetalins against rat aorta contraction induced by norepinephrine, some segetalines, i.e., segetalins F, G, and H, isolated from *Vaccaria segetalis*, showed relatively strong relaxant activity, and segetalin B exhibited contractile activity. This interesting finding was interpreted as a result of the basic Lys and Arg residues being present in segetalins F, G, and H, whereas in segetalin B, there is no basic residue; instead, a Trp residue is present (27, 30).

As cyclopeptides may be produced biosynthetically by ribosomal- or nonribosomal-dependent peptide synthases, some authors investigated the biosynthesis of Caryophyllaceae-like cyclopeptides in the developing seeds of *Saponaria vaccaria*. They found that there are genes to encode the precursors of cyclic peptides, which ultimately are cyclized and result in the final products. This finding was further confirmed by expressing a cloned DNA (cDNA) in the roots of transformed *S. vaccaria* to encode a synthetic precursor of segetalin A. During the investigation, the authors predicted the presence of two more segetalins, i.e., segetalins J and K in *S. vaccaria* seeds. The prediction by sequence analysis also revealed the existence of genes for encoding cyclic peptide precursors in *Dianthus caryophyllus* and *Citrus* species (33). The sequences of all segetalins are shown in **Table 1**.

## Yunnanins

**Figure 2:**
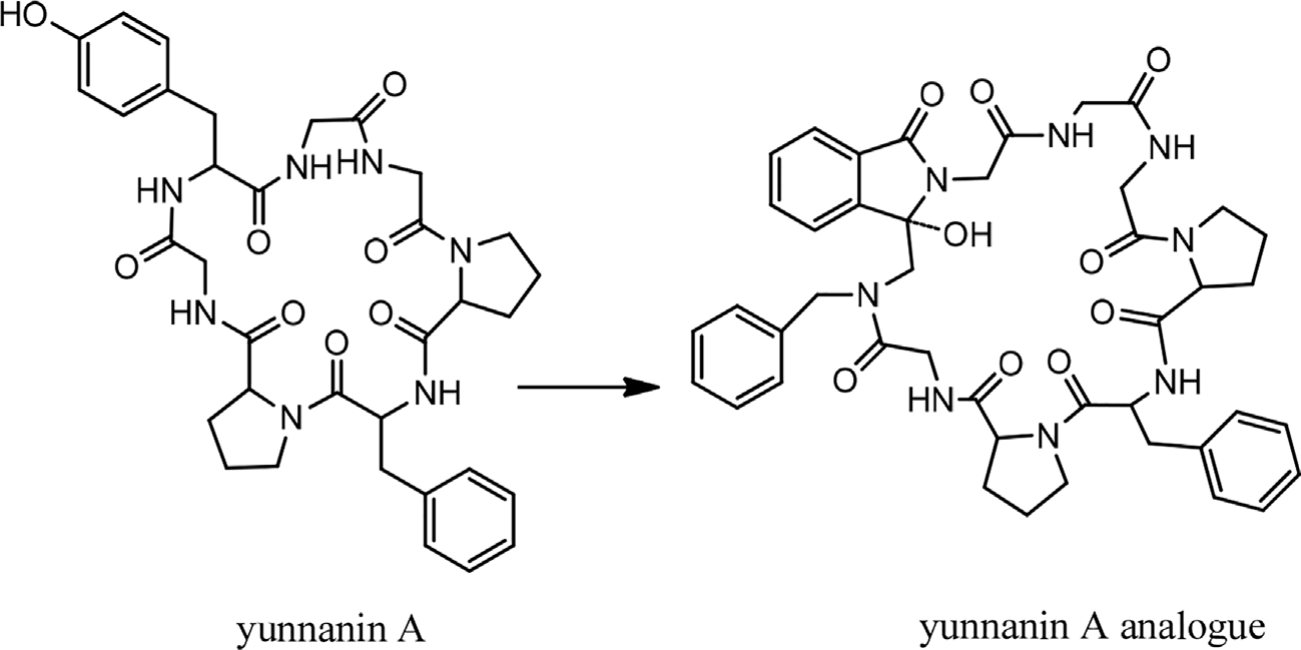
The structure of yunnanin A and its designed analogue.

**Chart 2:**
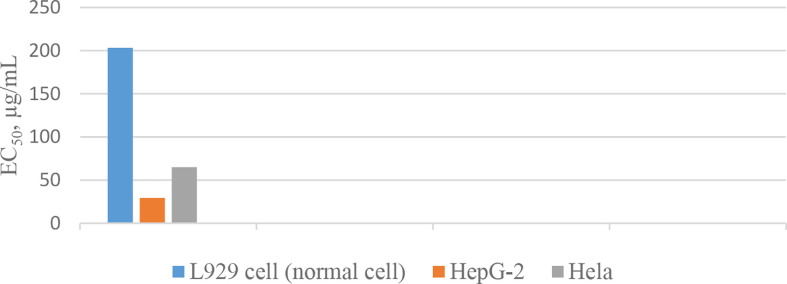
Inhibitory action of the yunnanin A analogue against cell lines.

In 1994, the isolation of two cyclic peptides, yunnanin A and B, was reported from the root of *Stellaria yunnanensis* (Caryophllaceae) (34). The peptide structures were confirmed by spectroscopic analysis and chemical methods (34, 35). Yunnanin A showed cytotoxic activity against p-338 cells (36). Yunnanin C, isolated from the root of the same plant (36), as well as yunnanin A, indicated *in vitro* antiproliferative activities against three cell lines—J774.A1 (murine monocyte/macrophage cell line), WEHI-164 (murine fibrosarcoma cell line), and HEK-293 (human epithelial kidney cell line)—after three days of incubation (*IC_50_* s ranging from 2.1 to 7.5 µg/mL) (37). Interestingly, the synthetic counterparts of these cyclopeptides did not show the same level of cytotoxicity against the above cell lines (*IC*_50_ s less than 100 µg/mL). This discrepancy was discussed as it might be as a result of subtle conformational changes of proline units during the synthesis process, which ended by diverse arrangements of proline residues in the synthetic cyclopeptides (37). In another experiment, the synthesized yunnanin A showed weak antimicrobial, anti-inflammatory, and anthelmintic activities (38). Recently, a cyclic analogue of yunnanin A was synthesized by eliminating tyrosine residue and introducing a phthalimide structure instead, inside the ring structure, through photo inducing a single electron transfer reaction (SET) (**Figure 2**). The hydroxyl group attached to the isoindolinone part of the phthalimide structure could have a role similar to the hydroxyl group of the eliminated tyrosine residue. It is shown that this analogue exhibits strong toxicity against HepG-2 and Hela cell lines (*IC_50_* s 29.25 µg/mL and 65.01 µg/mL, respectively) (**Chart 2**). This cytotoxicity had almost no toxic activity against normal cells, L929 cell lines (*IC_50_* 203.25 µg/mL) (**Chart 2**). It is suggested that special intramolecular hydrogen binding and γ, β–turn secondary structures of this cyclic peptide analogue could be possible reasons for producing such high activity (39).

**Figure 3:**
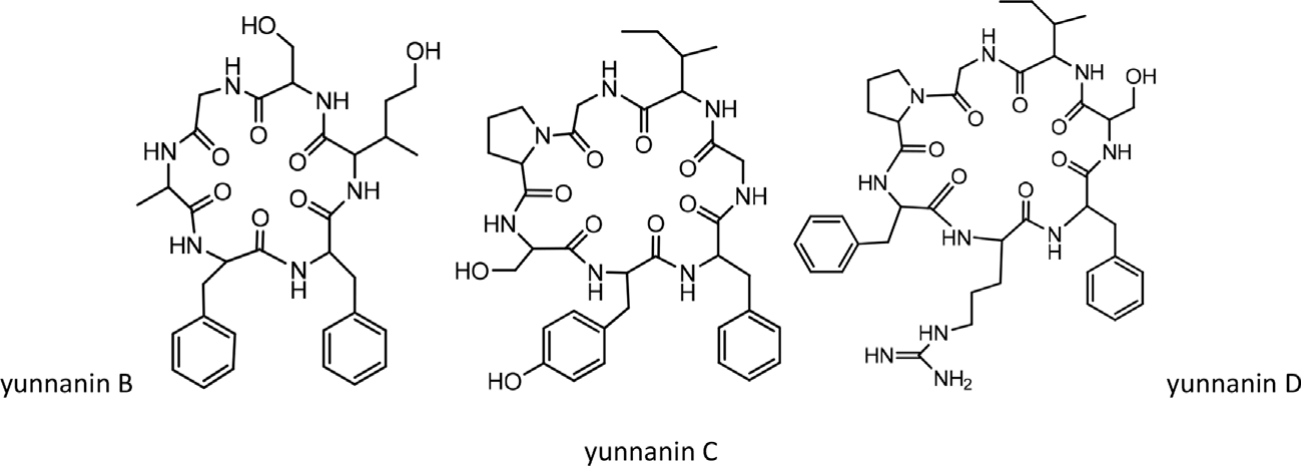
The structures of yunnanin B, C, D.

On the other hand, the synthesis of yunnanin C was also reconsidered along with the preparation of 9 related mutated analogues by the method of serine/threonine ligation (STL)–mediated cyclization (40). Three other yunnanins, named yunnanins D, E, and F, were isolated from *Stellaria* yunnanensis, and their structures were confirmed by spectroscopic and chemical analysis (41). Later, in 2005, the total synthesis of yunnanin F was reported using a disconnection approach (42). By screening for antimicrobial and pharmacological activities, yunnanin F showed moderate-to-good inhibitory activity against the growth of bacterial cells and weak activity against fungal cells (42). Meanwhile, this cyclopeptide demonstrated a good anthelmintic activity against earthworms (42). From the point of pharmacological activity, yunnanin F produced moderate anti-inflammatory activity (42). The structures of yunnanin B, C, and D are shown in **Figure 3**. The peptide sequences and information on biological activities of all the yunnanin cyclopeptides are given in **Table 2**.

**Table 2:** Yunnanin cyclopeptides with their sequences and biological activities.

Peptide names	Peptide sequence	Cytotoxic activity	Pharmacological activities	Anti-infective activities
Yunnanin A	Cyclo(-Gly-Tyr-Gly-Gly-Pro-Phe-Pro)	+	weak anti-inflammatory	weak antimicrobial, anthelmintic
Yunnanin B	Cyclo(-Gly-Ser-δ-HO Ile-Phe-Phe-Ala)	+		
Yunnanin C	Cyclo(-Gly-Ile-Gly-Phe-Tyr-Ser-Pro)	+		
Yunnanin D	Cyclo(-Gly-Ile-Ser-Phe-Arg-Phe-Pro)	+		
Yunnanin E	Cyclo(-Gly-Ser-δ-HO Ile-Phe-Phe-Ser)			
Yunnanin F	Cyclo(-Gly-Val-Thr-Trp-Tyr-Pro-Ser-Ser)		anti-inflammatory	Antimicrobial, anthelmintic

## Dichotomins

**Chart 3:**
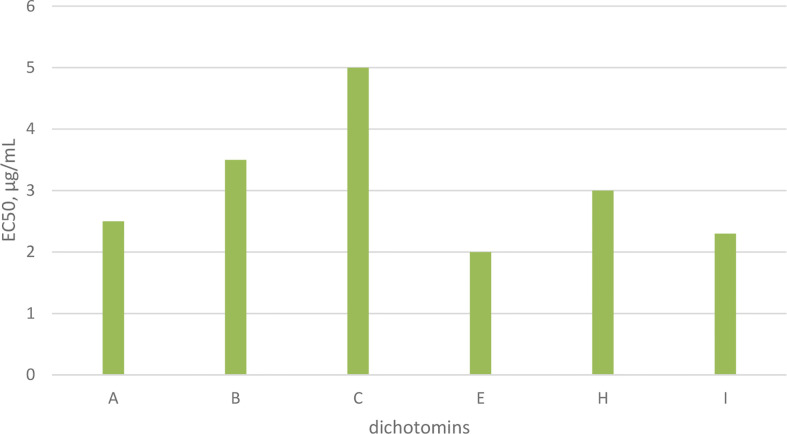
Anticancer activity of dichotomins on p-388 cells.

**Figure 4:**
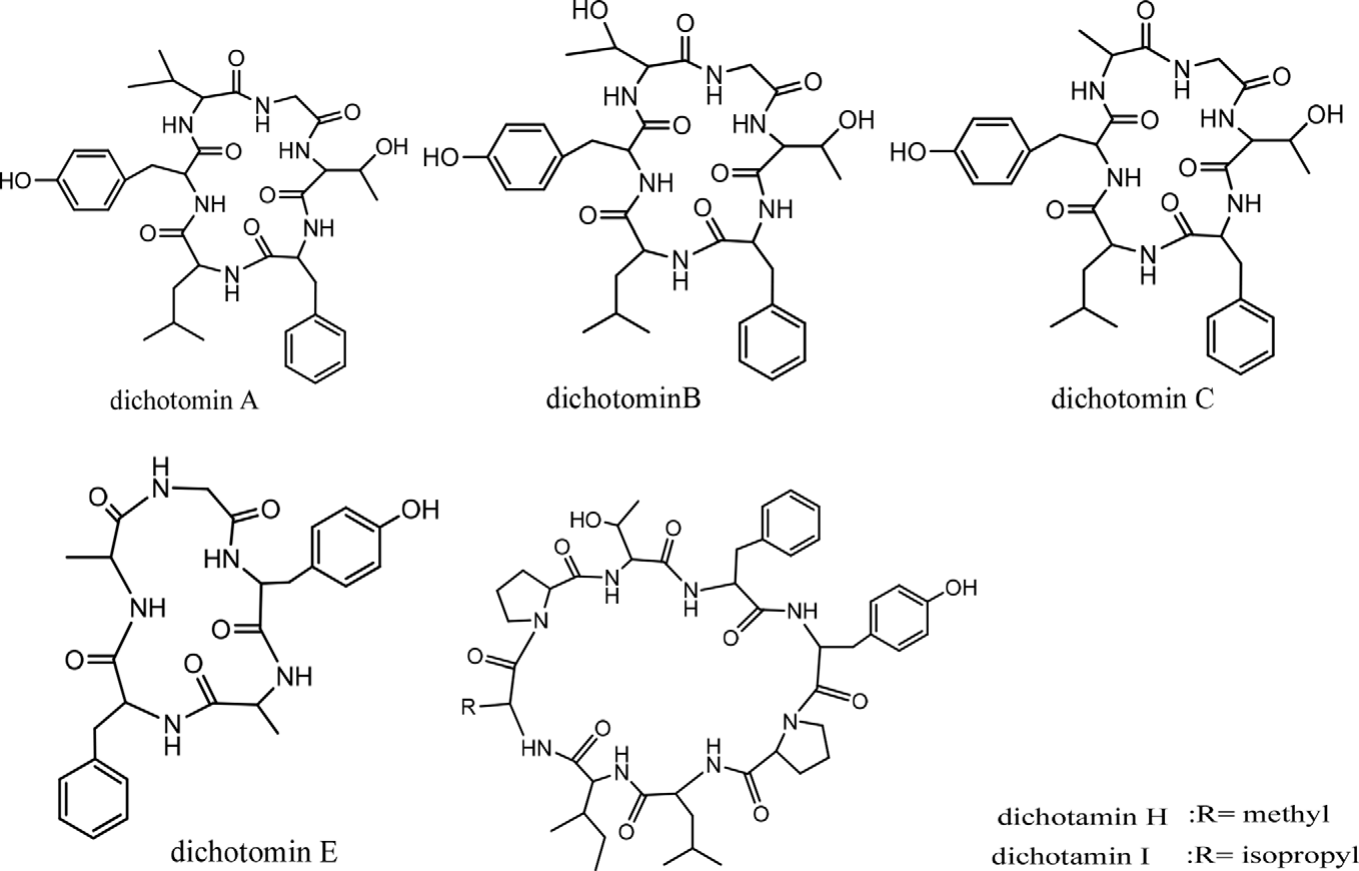
The structures of dichotamins.

Dichotomins A to E were isolated from the roots of *Stellaria dichotoma* L. vat. *lanceolata* Bge, and their structures were defined by 2D NMR spectroscopy, X-ray, and chemical analysis (43, 44). In addition, the biological activities of these cyclopeptides were also reported by the same authors. Dichotomins A, B, C, and E demonstrated inhibitory action against the growth of p-388 lymphocytic leukemia cells (*IC_5_*_0_ s were 2.5, 3.5, 5.0, 2.0 µg/mL, respectively) (**Chart 3**). Interestingly, dichotomin D did not show such activity. It is argued that dichotomins A, B, and C, as hexacyclopeptides, have identical sequences except at the sixth residues. To show similarity between dichotomin E, a pentacyclopeptide, with the three dichotomins A, B, and C, the sequence of Tyr-Ala-Phe in dichotomin E was compared with the sequence of Phe-Leu-Tyr in these hexacyclopeptides. By this comparison, a comment was made that a common feature exists as one aliphatic residue is present within two aromatic residues among A, B, C, and E cyclopeptides. Dichotomin D, as a hexacyclopeptide, had neither an identical sequence with the sequence of dichomins A, B, and C nor an aliphatic residue separating two aromatic residues. In fact, the two aromatic residues of dichotomin D were close together. Instead of cytotoxic activity, dichotomin D showed a strong cyclooxygenase inhibitory action (about 73% inhibition at 100 µM concentration compared with the control). Dichotomin A did not show activity against cyclooxygenase (44). Later on, dichotomins F and G were isolated from the same plant source, and their structures were confirmed by instrumental and chemical methods (45). Both dichotomin F and G demonstrated inhibitory action on cyclooxygenase. In another report, dichotomins H and I were found from the same plant, and their biological activities were studied (46). Using an MTT assay, dicotomins H and I were shown to have inhibitory activity against the growth of p-388 cells (*IC_50_* s 3.0 and 2.3 µg/mL, respectively) (**Chart 3**). In a further study, dichotomin J, naturally originated from the roots of *Stellaria dichotoma*, was synthesized and screened for antibacterial, antifungal, and anthelmintic activities and compared with the appropriate related standard drugs, such as ciprofloxacin, griseofulvin, and albendazole, respectively. It is shown that this cyclopeptide contains good inhibitory action against bacteria (*S. aureus*, *B. substilis*) and moderate activity against fungi (*C. albicans*, *A. niger*) in 50 µg/mL concentration and high killing activity against earthworm (27.65 min, mean death time comparable with 22.78 min mean death time found for albendazole) (47). The structures of anticancer dichotomins are shown in **Figure 4**. The peptide sequences and information on biological activities of all the dichotomin cyclopeptides are given in **Table 3**.

**Table 3:** Dichotomin cyclopeptides with their sequences and biological activities.

Peptide names	Peptide sequence	Cytotoxic activity	Pharmacological activities	Anti-infective activities
Dichotomin A	Cyclo(-Gly-Thr-Phe-Leu-Tyr-Val)	+		
Dichotomin B	Cyclo(-Gly-Thr-Phe-Leu-Tyr-Thr)	+		
Dichotomin C	Cyclo(-Gly-Thr-Phe-Leu-Tyr-Ala)	+		
Dichotomin D	Cyclo(-Gly-Val-Gly-Phe-Tyr-Ile)		Cyclooxygenase inhibitor	
Dichotomin E	Cyclo(-Gly-Tyr-Ala-Phe-Ala)	+		
Dichotomin F	Cyclo(-Pro-Tyr-Phe-Val-Leu-Pro-Ser-Val-Tyr)		Cyclooxygenase inhibitor	
Dichotomin G	Cyclo(-Pro-Leu-Pro-Ile-Pro-Pro-Phe-Tyr-Ser)		Cyclooxygenase inhibitor	
Dichotomin H	Cyclo(Ala-Pro-Thr-Phe-Tyr-Pro-Leu-Ile)	+		
Dichotomin I	Cyclo(-Val-Pro-Thr-Phe-Tyr-Pro-Leu-Ile)	+		
Dichotomin J	Cyclo(-Gly-Ile-Phe-Leu-Tyr-Ala)			antibacterial, antifungal, anthelmintic

## Cycloleonuripeptides

**Chart 4:**
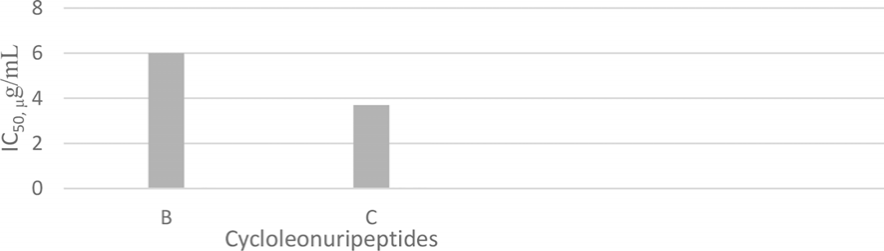
Inhibitory activity of cycloleonuripeptides on p-338 lymphocytic leukemia cells.

**Figure 5:**
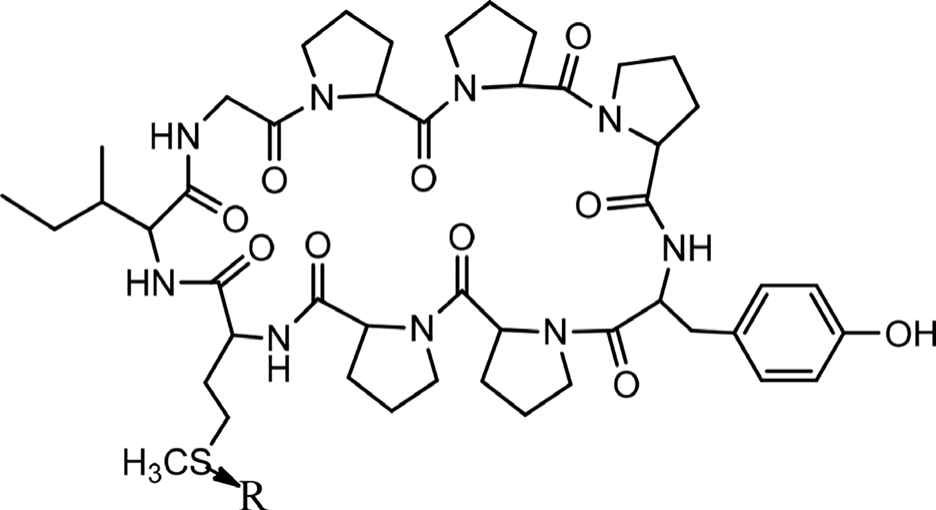
The structures of cycloleonuripeptides A, (B,C) (R=O). B and C are isomers.

Cycloleonuripeptides as proline-rich cyclopeptides were discovered from the fruits of *Leonurus heterophyllus* (Labiatae). Cycloleonuripeptides A, B, and C are nonacyclopeptides (**Figure 5**). Among them, cycloleonuripeptide B is epimer with cycloleonuripeptide C. The stuctures of these cyclopeptides were elucidated by 2D NMR and chemical analysis (48). The presence of proline residues in the peptide backbone could end with several possible conformations as a result of cis-trans isomerization of amide bonds involving proline. Therefore, conformational analysis of the cyclopeptides were carried out by distance geometry calculation and restrained energy minimization using NMR data. Although five proline residues are present in these peptide sequences, a single stable conformer was observed for them. In addition, it was found that the skeleta of cycloleonuripeptides A, B, and C contain two β-turns (49). Cycloleonuripeptides B and C exhibit inhibitory action on the growth of p-338 lymphocytic leukemia cells (*IC_50_*s 6.0 and 3.7 µg/mL, respectively) (**Chart 4**) (48). Further experiments on the fruit extract of *Leonurus heterophyllus* resulted in the discovery of cycloleonuripeptides D (50), E, and F (51). Cycloleonuripeptide D shows inhibitory action against a cyclooxygenase enzyme, and cycloleonuripeptides E and F demonstrate moderate activities as vasorelaxant agents in rat aorta (50, 51). The peptide sequences and information on biological activities of all the cycloleonuripeptides are given in **Table 4**.

**Table 4:** The sequences of Cycloleonuripeptide cyclopeptides and related biological activities.

Peptide names	Peptide sequence	Cytotoxic activity	Pharmacological activities
Cycloleonuripeptide A	Cyclo(-Gly-Pro-Pro-Pro-Tyr-Pro-Pro-Met-Ile)		
Cycloleonuripeptide B	Cyclo(-Gly-Pro-Pro-Pro-Tyr-Pro-Pro-Met(O)-Ile)	+	
Cycloleonuripeptide C	Cyclo(-Gly-Pro-Pro-Pro-Tyr-Pro-Pro-Met(O)-Ile)	+	
Cycloleonuripeptide D	Cyclo(-Ser-Pro-Pro-Pro-Tyr-Phe-Gln-Thr-Pro-Ile)		Cyclooxygenase inhibitor
Cycloleonuripeptide E	Cyclo(-Ala-Pro-Ile-Val-Ala-Ala-Phe-Thr-Pro)		Vasorelaxant
Cycloleonuripeptide F	Cyclo(-Gly-Tyr-Pro-Leu-Pro-Phe-Tyr-Pro-Pro)		Vasorelaxant

## Cyclolinopeptides

**Chart 5:**
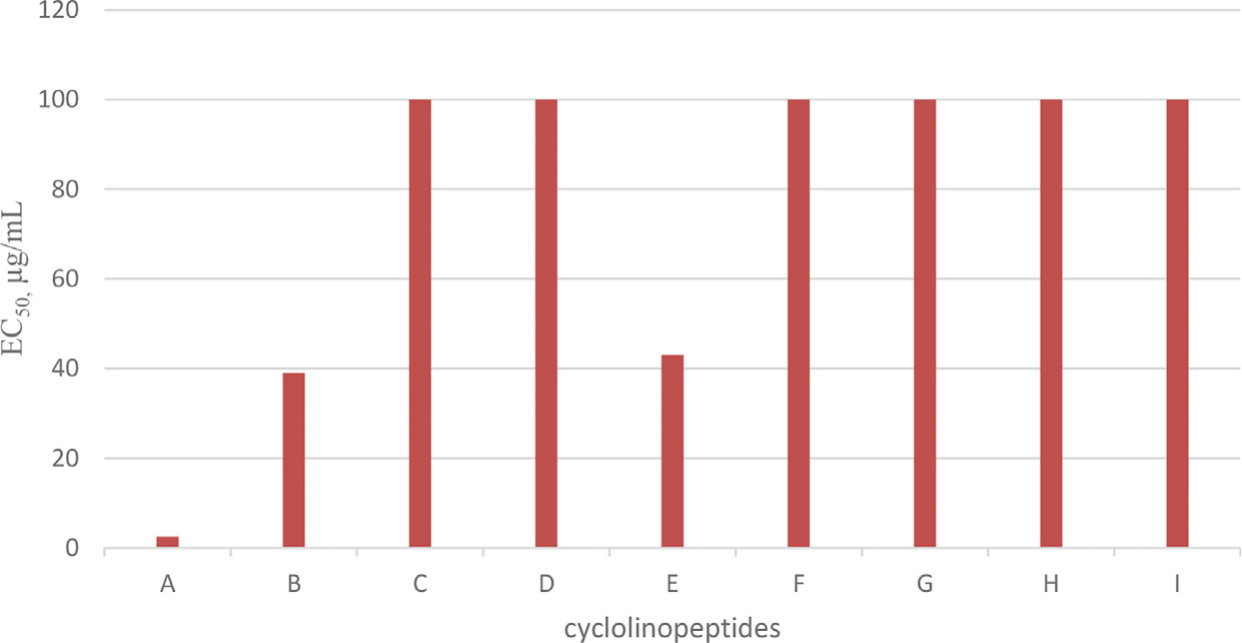
Antiproliferative activity of cyclolinopeptides on mouse lymphocyte cells.

**Figure 6:**
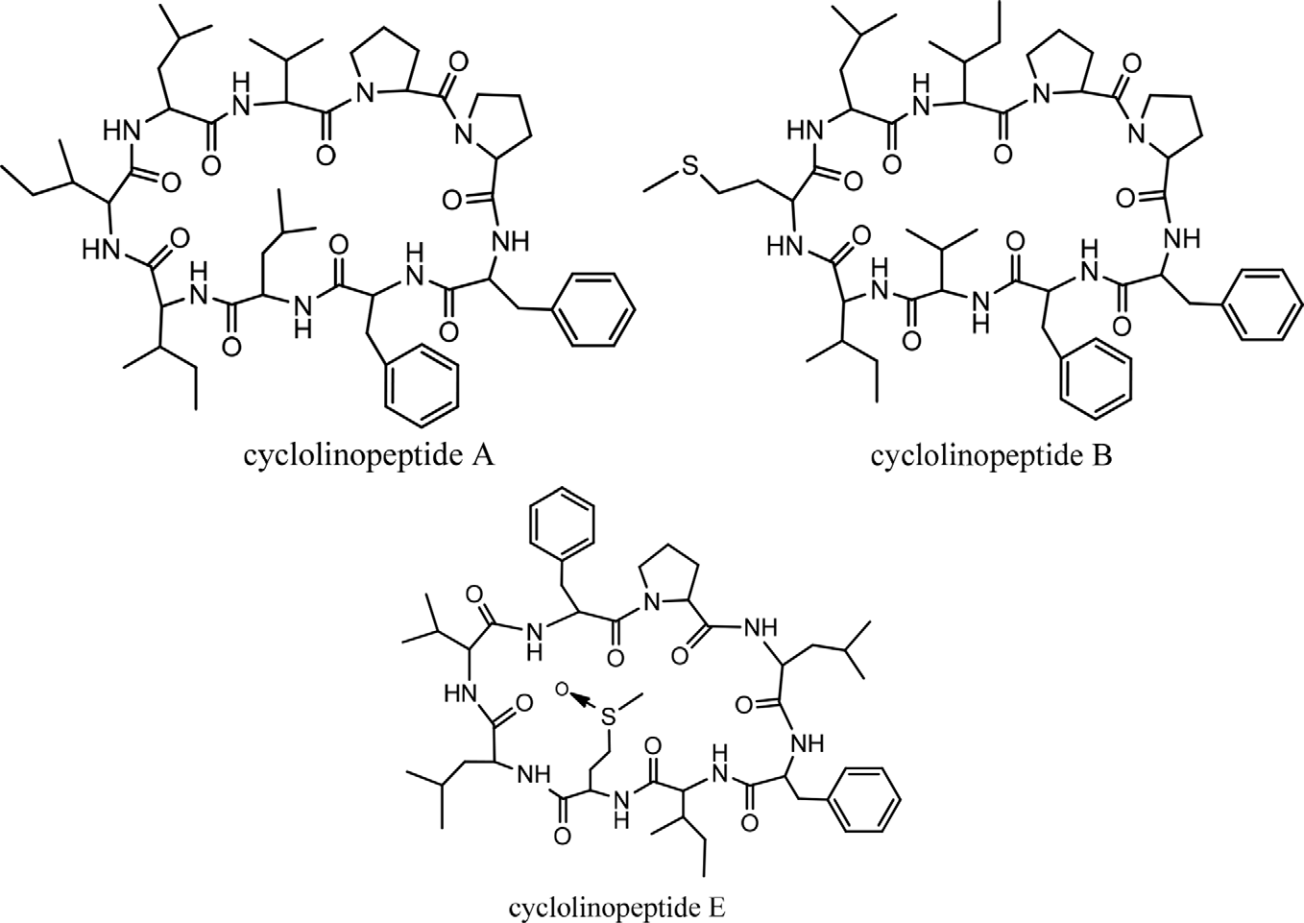
The structures of cyclolinopeptides.

Cyclolinopeptide A (CLA), as a first natural cyclopeptide, was isolated from the seeds of *Linum usitatissimun* (linseed oil) in 1959 (52). Conformational analysis by NMR shows that cyclolinopeptide A occurs as at least four types of conformers in solution state, and none of them contains hydrogen binding intramolecularly. Therefore, high flexibility of the peptide molecule can result in solution (53). This peptide is active as a potent immunosuppressive compared with cyclosporine A (23). The search for more cyclolinopeptides was followed by the discovery of cyclolinopeptides B–E, and their structures were elucidated by 2D NMR and chemical analysis (54). Studying the biological activities of these cyclopeptides showed that cyclolinopeptide B possesses inhibitory activity against concanavalin A-induced mitogenic response of human peripheral blood lymphocytes (*IC_50_*44 ng/mL), comparable with cyclosporine A (55). Cyclolinopeptides A, B, and E also show moderate inhibition on the proliferation of mouse lymphocyte cells induced by concanavalin A (*IC_50_* s 2.5, 39, and 43 µg/mL, respectively) (**Chart 5**) (54). In contrast, cyclolinopeptides C and D do not give such a level of activity (*IC_50_* >100 µg/mL). In another study, four cyclolinopeptides F–I were found in the seeds of *Linum usitatissimun.* The structures of isolated cyclopeptides were determined by instrumental and chemical methods. In addition, the immunosuppressive activity of these compounds was evaluated against mouse splenocytes (56). Cyclolinopeptides F–I also do not show immunosuppressive activity (*IC*_50_ >100 µg/mL). For these different results between cyclolinopeptides A, B, and C on one hand and cyclolinopeptides C, D, and F–I on the other, it is inferred that the biological activity is very dependent on the sequence as well as conformation of the peptides. To confirm this hypothesis, the three-dimensional structure of cycylolinopeptide A, studied by X-ray, and its distance geometry calculations were compared with that of cyclolinopeptide B. It was found that, in the solid state, conformation of cyclolinopeptide A was similar to that of the both cyclopeptides A and B in the solution state (57). The structures of cytotoxic cyclolinopeptides are shown in **Figure 6**. The peptide sequences and information on biological activities of all the cyclolinopeptides are given in **Table 5**.

**Table 5:** The sequences of cyclolinopeptides and their biological activities.

Peptide names	Peptide sequence	Cytotoxic activity	Pharmacological activities
Cyclolinopeptide A	Cyclo(-Pro-Pro-Phe-Phe-Leu-Ile-Ile-Leu-Val)	+	immunosuppressive
Cyclolinopeptide B	Cyclo(-Pro-Pro-Phe-Phe-Val-Ile-Met-Leu-Ile)	+	immunosuppressive
Cyclolinopeptide C	Cyclo (-Pro-Pro-Phe-Phe-Val-Ile-Met(O)-Leu-Ile)		
Cyclolinopeptide D	Cyclo(-Pro-Phe-Phe-Trp-Ile-Met(O)-Leu-Leu)		
Cyclolinopeptide E	Cyclo(-Pro-Leu-Phe-Ile-Met(O)-Leu-Val-Phe)	+	immunosuppressive
Cyclolinopeptide F	Cyclo(-Pro-Phe-Phe-Trp-Val-Met(O)-Leu-Met(O)		
Cyclolinopeptide G	Cyclo(-Pro-Phe-Phe-Trp-Ile-Met(O)-Leu-OMet)		
Cyclolinopeptide H	Cyclo(-Pro-Phe-Phe-Trp-Ile-Met(O)-Leu-Met)		
Cyclolinopeptide I	Cyclo(-Pro-Phe-Phe-Trp-Val-Met-Leu-Met(O))		

## Cherimolacyclopeptides

**Figure 7:**
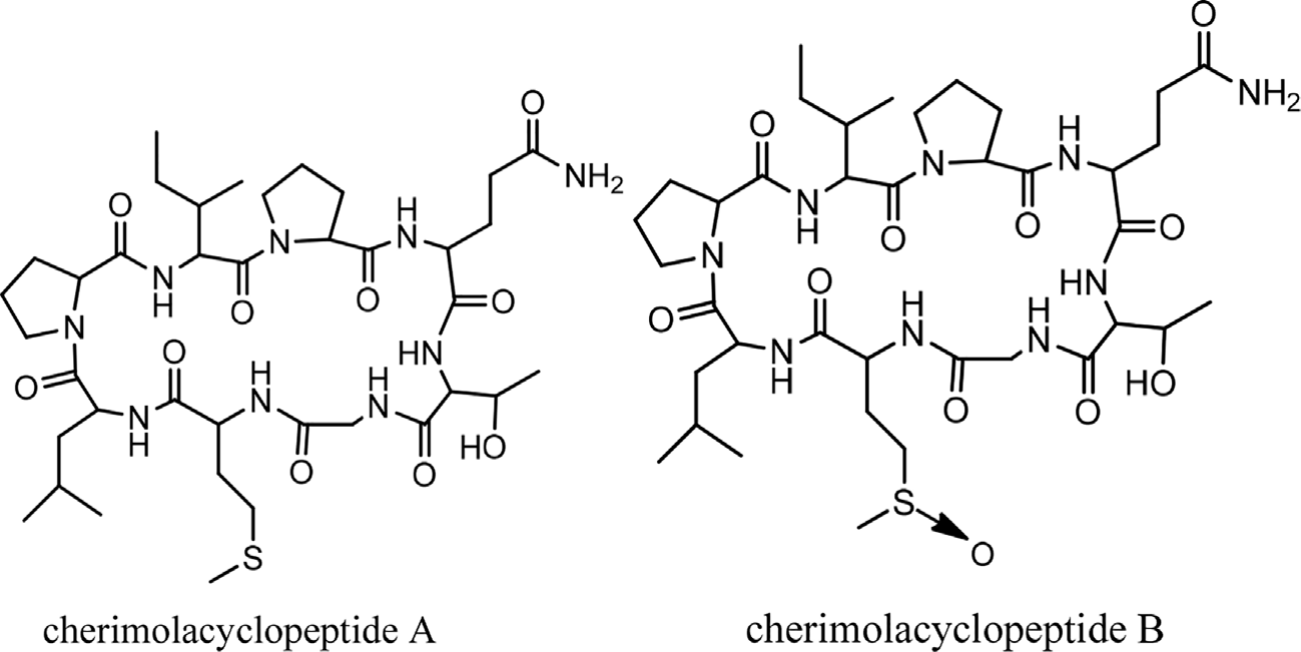
The structures of cherimolacyclopeptides.

**Chart 6:**
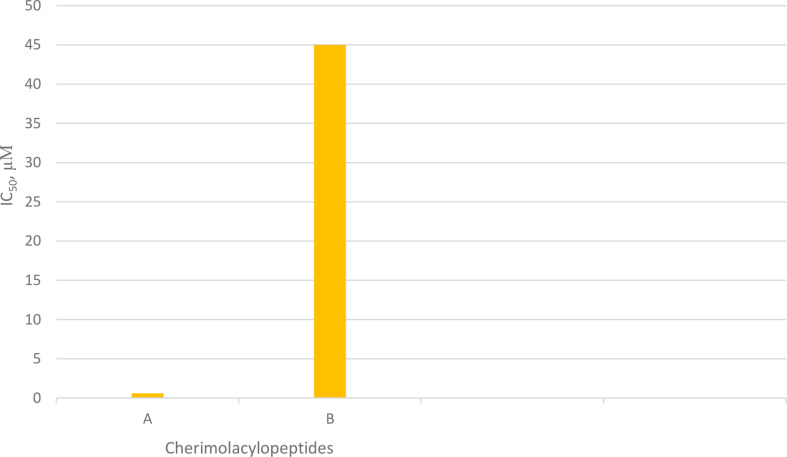
Cytotoxic activity of cherimolacylopeptides on KB tumor cells.

Cherimolacyclopeptides A and B were isolated from the seeds of *Annona cherimola* Miller. Tandem mass spectrometry and 2D NMR spectroscopy were used to determine the peptide sequences (**Figure 7**). The solution state structure of cherimolacyclopeptide A was also studied. It is shown that this cyclopeptide contains two β-turns and one new type of β-bulge compared with other cyclopeptides. A cytotoxicity study showed that cherimolacyclopeptide A is a potent cytotoxic agent (*IC_50_* 0.6 µM), but cherimolacyclopeptide B is a weak cytotoxic agent against KB tumor cells (*IC_5_*_0_ 45 µM) (**Chart 6**) (58). The peptide sequences and information on biological activities of cherimolacyclopeptides are given in **Table 6**.

**Table 6:** The cherimolacyclopeptides, their sequences, and biological activities.

Peptide names	Peptide sequence	Cytotoxic activity
Cherimolacyclopeptide A	Cyclo(-Pro-Gln-Thr-Gly-Met-Leu-Pro-Ile)	+++ (high potency)
Cherimolacyclopeptide B	Cyclo(-Pro-Gln-Thr-Gly-OMt-Leu-Pro-Ile)	+ (low potency)

## Dianthins

*Dianthus* is a genus of *Caryophyllaceous* that includes 300 species (59). From some *Dianthus* species, three classes of compounds have been isolated and studied; 1) triterpenoid saponins, called dianosides A and B (60), C–F (61), and G–I (62); 2) dianthin proteins dianthin-30 and dianthin-32 (63) and dianthin-29 (64); and 3) dianthin cyclic peptides A and B (65), C–F (59), G and H (66), and I (67, 68). It should be noted that, because proteins and cyclopeptides of *Dianthus* have the common name of dianthin, these two different classes must not be confused. In this review, isolation, synthesis, and biological activities of dianthin cyclopeptides are discussed. Discussion of the biological activities is focused on the anticancer activity of these cyclopeptides.

### Dianthin Isolation and Synthesis

#### Isolation

**Figure 8:**
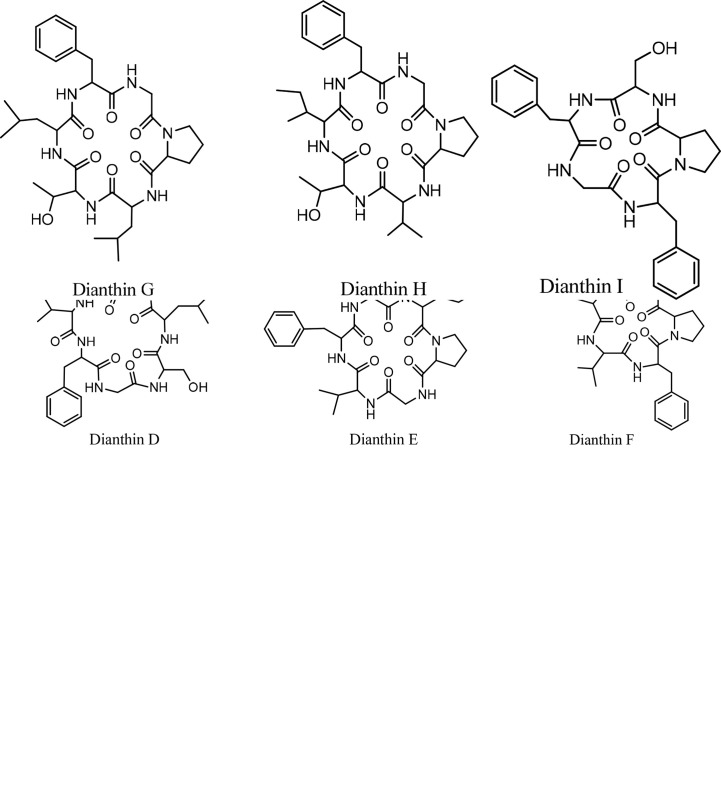
The structures of Dianthins.

**Figure 9:**
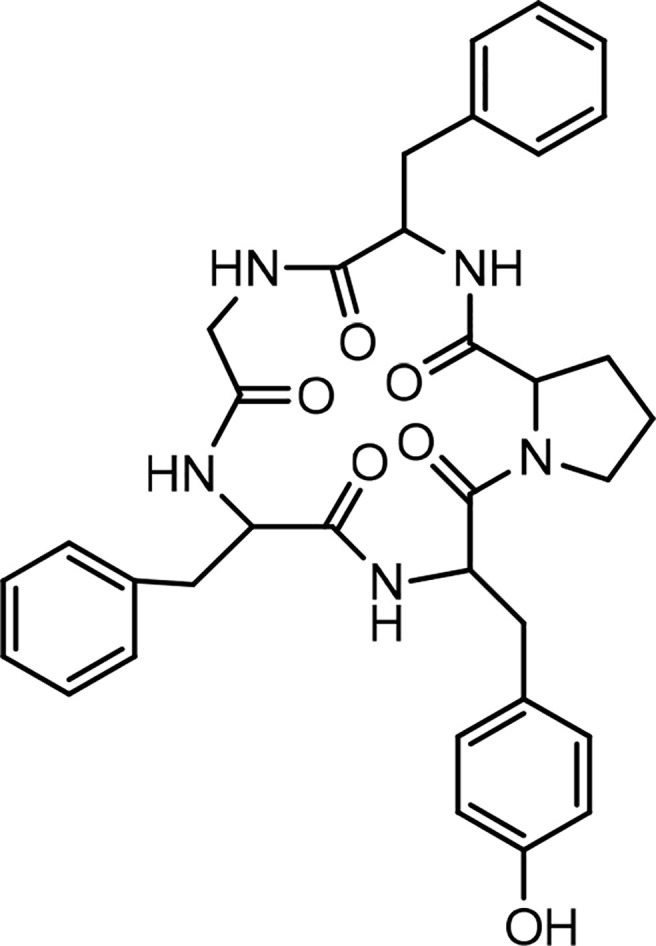
Structure of Longicalycinin A.

Historically, the plant *Dianthus superbus* L. has been used in China as a traditional medicine for its several biological activities, including diuretic, anti-inflammatory, urinary anti-infective, and anticancer effects (69). In an initial study, two cyclopeptides were isolated from this plant, and their structures were determined as cyclo(-Ala-Tyr-Asn-Phe-Gly-Leu) (dianthin A) and cyclo(-Ile-Phe-Phe-Pro-Gly-Pro) (dianthin B), using instrumental analysis (65). In the following study, four cyclopeptides, dianthins C, D, E, and F, are isolated from the extract of the same plant, and their structures are documented by mass spectrometry, 2D NMR analysis, and some chemical methods (59). In this study cytotoxicity of dianthin E is also reported. In another study, two other cyclopeptides are identified from the extract of *Dianthus superbus* by employing ESI tandem mass fragmentation, 2D NMR analysis, and X-ray diffraction (28). The proliferative activities of dianthins G and H are also evaluated in this study. A few years ago, the isolation of a new cyclopeptide named dianthin I was reported from *Dianthus chinensis*, and its structure was determined (67). From *Dianthus superbus* var. *longicalysinus*, along with six other known compounds, a cyclopeptide called longicalycinin A was isolated and its structure identified by instrumental analysis and reported as cyclo(-Gly-Phe-Tyr-Pro-Phe) (**Figure 1**). The biological evaluation of this compound shows its cytotoxicity against the HepG2 cancer cell line (70). So far, there are no other reports on the discovery of new longicalycinin although total synthesis of this and some other cyclopeptides from *Dianthus superbus* has been documented in several studies (71–75). The structures of dianthins and longicalycinin A are presented in **Figures 8** and **9**. The peptide sequences and information on biological activities of dianthins and longicalycinin A are given in **Table 7**.

**Table 7:** Dianthins, their sequences, and biological activities.

Peptide names	Sequence	Anticancer activity	Pharmacological activities	Anti-infective activities
Dianthin A	cyclo-(Ala-Tyr-Asn-Phe-Gly-Leu)	+		Anthelmintic Antifungal
Dianthin B	cyclo-(Ile- Phe- Phe-Pro- Gly-Pro)			
Dianthin C	cyclo-(Gly-Pro-Phe-Tyr-Val-Ile)	+		
Dianthin D	cyclo-(Gly-Ser-Leu-Pro-Pro-Ile-Phe)	+		
Dianthin E	cyclo-(Gly-Pro-Ile-Ser-Phe-Val)	+	Proliferation stimulant	
Dianthin F	cyclo-(Gly-Pro-Phe-Val-Phe)	+		
Dianthin G	cyclo-(Gly-Pro-Leu-Thr-Leu-Phe)		Proliferation stimulant	
Dianthin H	cyclo-(Gly-Pro-Val-Thr-Ile-Phe)	+	Proliferation stimulant	
Dianthin I	cyclo-(Gly–Phe–Pro–Ser–Phe)			
Longicalycinin A	cyclo-(Gly-Phe-Tyr-Pro-Phe).	+		anthelmintic

#### Synthesis of Dianthins

Dianthin A, also called cyclopolypeptide (XIII), was synthesized through several chemical steps using a solution phase peptide synthesis strategy (71). The synthesis of dianthin I by the solid phase method was reported by a group of scientists (68). The synthesis of longicalycinin A was also reported through solution phase (72) as well as solid phase methods (73, 74). In addition, several analogues of longicalycinin A were synthesized in order to achieve information about relationships between structure and activity of this cyclopeptide (74). For the synthesis of longicalycinin A analogues, a two-step synthesis strategy was chosen. To explain more, linear peptides at first were synthesized on 2-chlorotrityl (2-CTC) resin and then detached from the resin as protected peptides in the partial cleavage step. In the second step, final deprotection was applied in the solution phase in order to achieve fully unprotected linear peptides. For preparing cyclic peptide analogues, after cleaving protected linear peptides from the resin, conditions for peptide cyclization were employed in the solution phase followed by final deprotection. The reaction product was then solidified in cold diethyl ether. In the other effort, linear and cyclic heptapeptide analogues of longicalycinin A were also synthesized by employing two cysteine molecules (75). The cysteine molecules were added as one at the C-terminal and the other at the N-terminal position of the longicalycinin A linear peptide analogues. In this experiment, the peptide cyclization was performed in an oxidation condition in order to make a disulfide bond between two SH groups of cysteine residues of the linear peptides.

#### Biological Activities of Dianthin Cyclopeptides

Various biological activities have been reported in the literature concerning dianthins A–I and longicalycinin A. The studies were made on the evaluation of antiprotozoal, antifungal, antiduretic, anti-inflammatory, and anticancer activities of these cyclopeptides. The survey of these studies is presented as follows.

### Anticancer Activity of Dianthins

**Chart 7:**
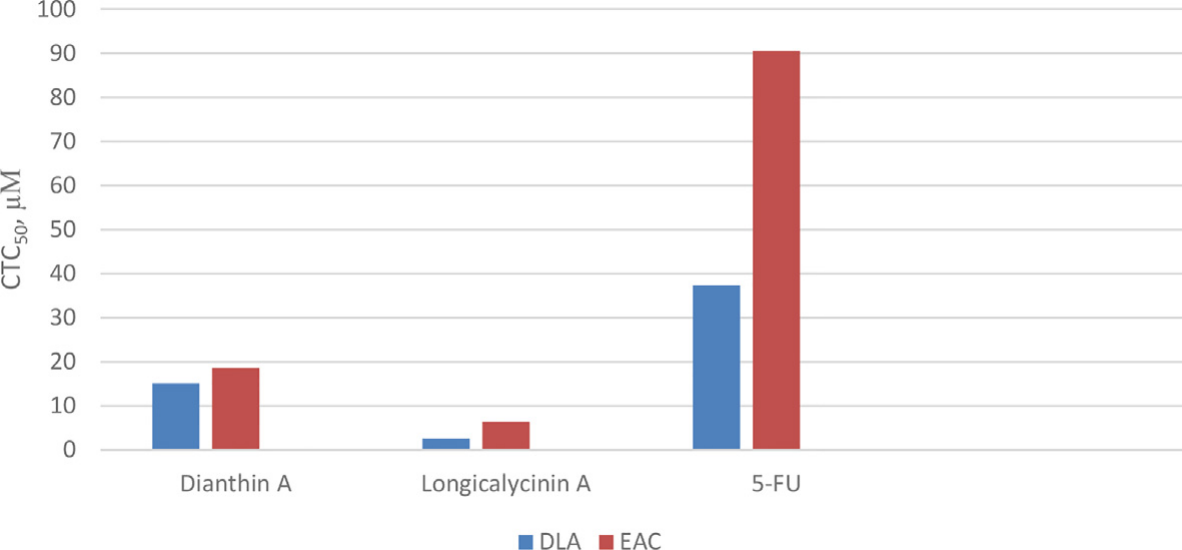
Cytotoxicity of longicalycinin A, compared with dianthin A and 5- Fluorouracil (5-FU) as standard drug on cancer cells, Dalton’s lymphoma ascites (DLA) and Ehrlich’s ascites carcinoma (EAC).

**Chart 8:**
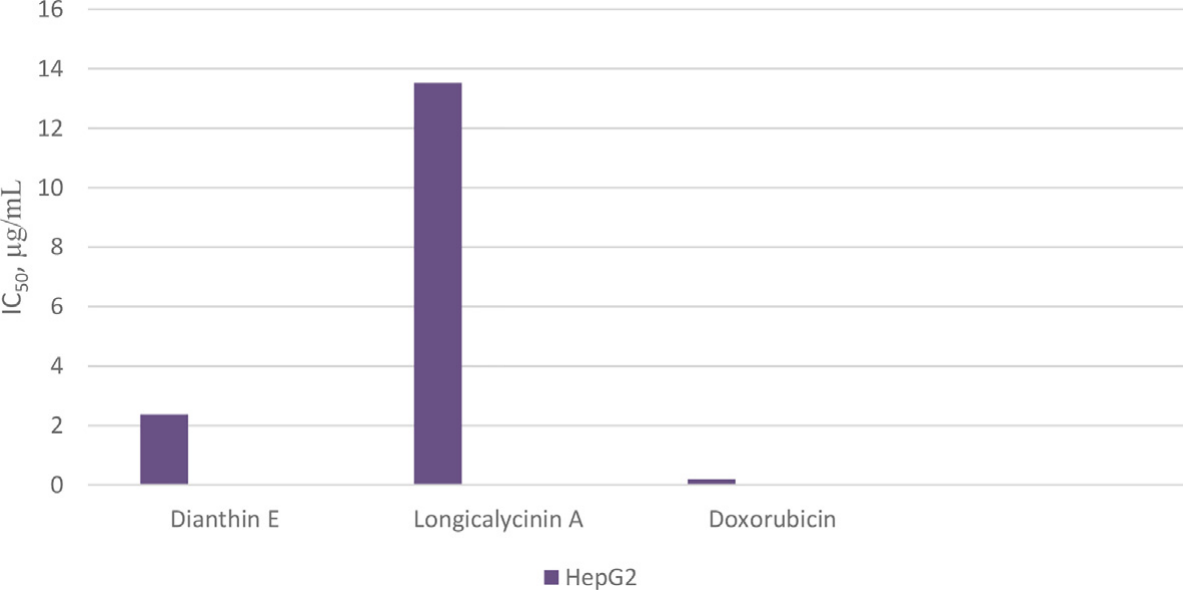
Cytotoxicity of longicalycinin A on cancer cells, HepG2, compared with dianthin E and doxorubicin (as standard drug).

As previously mentioned, *Dianthus superbus*, a genus of plant family *Caryophyllaceae*, has been used for anticancer activity (69, 70). In one study, the antioxidant activity (free radical scavenging ability) and cytotoxic effect of several fractions from an ethanol extract of *Dianthus superbus* on three human cancerous cell lines, HepG2, HeLa, and Bel-7402, were reported (76). It was demonstrated that, among these fractions, the ethyl acetate part containing a high content of phenolic compounds with high reducing ability showed the most antioxidant activity. Using the MTT assay, the ethyl acetate part also showed considerable cytotoxicity (*IC_50_* 20–36 µg/mL) against the three cell lines. In the other study, by employing the ethyl acetate fraction, apoptotic activation was observed in the HepG2 cell line (77). Treating with 80 µg/mL of the fraction over 24 h caused a considerable increase in the percentage of cells in the sub-G1 phase in which a high amount of apoptotic nuclear fragment bodies was seen. In a further experiment exposing HepG2 cells to the ethyl acetate fraction for 48 h, it was shown that the expressions of Bcl-2 and NF-κB were suppressed, and the amount of cytochrome c was increased in cytosol due to the release from mitochondria. Caspases-9 and -3 were also activated. All these data infer that the ethyl acetate fraction of the ethanol extract of *Dianthus superbus* induces apoptotic phenomena in Hep-G2 cells by a mitochondrial intrinsic pathway (77). Also, there are several reports considering the anticancer activities of dianthin cyclopeptides originally isolated from *Dianthus superbus.* In one study, the biological activities of the synthesized dianthin A, previously isolated from the whole plant of *Dianthus superbus*, are reported (71). Dianthin A shows a noticeable cytotoxic activity against two cancer cell lines, DLA and EAC cells (cytotoxic concentration inhibitory of 50% growth as *CTC_50_*, 15.1 and 18.6 µM, respectively) (**Chart 7**). 5-Fluorouracil (5-FU), as a standard drug, shows *CTC_50_*, 37.36 and 90.55 µM against the two cell lines, respectively (71). In another study, MTT as a cytotoxicity assay was used to examine the anticancer activity of all the compounds isolated from the methanol extract of *Dianthus superbus* (59). By employing different cancerous cell lines, Hep G2, Hep 3B, A-549, MCF-7, and MDA-MB-231, it was shown that, among dianthins C–F, only cyclopeptide E was effective against HepG2 with *IC_50_*, 2.37 µg/mL (**Chart 8**). Doxorubicin was chosen as the control drug in this experiment (*IC_50_*, 0.19 µg/mL) (59). Interestingly, by the MTT assay, it was shown that dianthins G, H, and E increased cell division (proliferation) of rat osteoblast cells, MC3T3-E1 *in vitro* (66). The highest cell stimulation activity for all three compounds was achieved at 1×10^-5^ mM. It was, therefore, suggested that these cyclic peptides may be potential candidates for osteoporosis therapy. In one study, by isolating different compounds from *Dianthin superbus* var. *longicalysinus*, only longicalycinin A, as a cyclopeptide, showed cell toxicity against HepG2 with *IC_50_* value 13.52 µg/mL by MTT assay (**Chart 8**) although several cell lines were employed, including human hepatocellular carcinoma Hep G2, Hep 3B, human breast carcinoma MCF-7, MDA-MB-231, and human lung carcinoma A-549 (70). The cytotoxic activity of longicalycinin A was also examined against DLA (NCRC 101) and EAC (NCRC 69) cell lines. 5-FU was used as the reference drug. Cytotoxic concentration of longicalycinin A for 50% growth inhibition (*CTC_50_*), determined by a graphical extrapolation method, was calculated as 2.62 and 6.37 µM for DLA and EAC cell lines, respectively (**Chart 7**) (72). The standard drug reported was also 5-FU with the previous *CTC_50_* reports (71). In a recent publication, longicalycinin A and its several analogues were synthesized, and their cytotoxic activities were examined against HepG2 and HT-29 using different experiments, including MTT, flow cytometry analysis, and Lysosomal membrane integrity assays. The results show that the two cyclopeptide analogues of longicalycinin A, cyclo-(Thr-Val-Pro-Phe-Ala), and cyclo-(Phe-Ser-Pro-Phe-Ala) were effective cytotoxic agents that were even better than longicalycinin A (74). Cytotoxic activity of linear and cyclic heptapeptide analogues of longicalycinin A containing two cysteine residues were also examined on the two cell lines HepG2 and HT-29, using MTT assay and flow cytometry analysis. Skin fibroblast cells were included in the experiment to evaluate if any toxicity of these peptides occurs on normal cells. As a cytotoxic reference, 5-FU was chosen (75). The result of the MTT assay show that the cyclic heptapeptide analogue was toxic against the cancer cell lines more than the linear heptapeptide congener. In addition, flow cytometry analysis demonstrates that apoptosis of the cancer cells can occur by the cyclic heptapeptide in higher percentages than by the linear heptapeptide analogue. In fact, the linear peptide showed no harmful effect on cancer cells as well as on skin fibroblast cells, whereas the cyclic heptapeptide could impose an apoptotic event (about 90%) on all the cancerous and fibroblast cells (75). As a conclusion from this experiment, further and better designed heptapeptide analogues of longicalycinin A containing a disulfide bond are needed to differentiate toxicity between cancer cells and normal cells. In the later study, various fractions of methanol extract of *Dianthus superbus*, i.e., ethyl acetate, butanol, and distilled water fractions as well as their bioactive compound content were evaluated *in vitro* from the points of antioxidant, anti-influenza, and cell toxicity activities (78). A cytotoxic activity assay against four cell lines, Hela (ovarian cancer cell), SKOV (ovarian cancer cell), Caski (cervical cancer cell), and NCL-H1299 (human lung cancer cell) showed that the ethyl acetate fraction was the most potent part of the methanol extract (with *IC_50_* s 9.5, 9.6, 13.8, and 69.9 µg/mL against the four cell lines, respectively). Because SKOV cells, as the most resistant ovarian cells, do not respond to the usual anticancer drugs such as Adriamycin and cis-platin, these results could be interesting. The authors show that the cyclic peptide content of the ethyl acetate extract was more than that of the other extracts. The butanol extract is shown to be of secondary importance with fewer phenolic compounds and, thus, with less cyclic peptide content. Moreover, dianthines C, D, H, and F, identified in the extracts by LC-MS/MS and measured by the correlation study, are shown to have cytotoxicity effects in the range of 75.7% to 99.7% (78).

### Other Biological Activities of Dianthins

Various species of *Dianthus* have been used in China, Korea, Iran, and Mongolia (78) for medicinal purposes other than carcinoma (65, 79). In one report, *Dianthus superbus* is shown to enhance cognition and improve memory in memory impaired mice under scopolamine induction. Thus, it is suggested that the plant could be useful in preventing Alzheimer disease (80).

*Dianthus superbus* has been also considered as a source of antioxidants for scavenging reactive oxygen radical species (ROS) (76, 81). That is because it contains phenolic compounds with high reducing power. These phenolic compounds mainly correlate with cyclic peptides’ content of the plant. In fact, antimicrobial and cytotoxic activities of *Dianthus superbus* may be somehow in parallel with the kinds of dianthin cyclopeptides present in the plant (76, 78, 81). On the other hand, antiviral activity of *Dianthus superbus* has been also reported against influenza viruses, but this activity corresponded to the presence of flavonol glycosides detected in the butanol fraction of the methanolic extract of the plant (78).

There are reports concerning the utilization of dianthins as antiparasitic agents (71, 72). Dianthin A is shown to have a strong antifungal activity against *Candida albicans* (MIC, 6 µg/mL) compared with griseofulvin. This cyclopeptide also demonstrated a moderate anthelmintic activity on earthworms in 2 mg/mL concentration, using membendazole/piperazine citrate as the standard drugs (71). A good anthelmintic activity of longicalycinin A was also reported in the literature (72). In addition, longicalycinin A showed moderate activity against dermatophytes (72).

## Concluding Remarks

**Figure 10:**
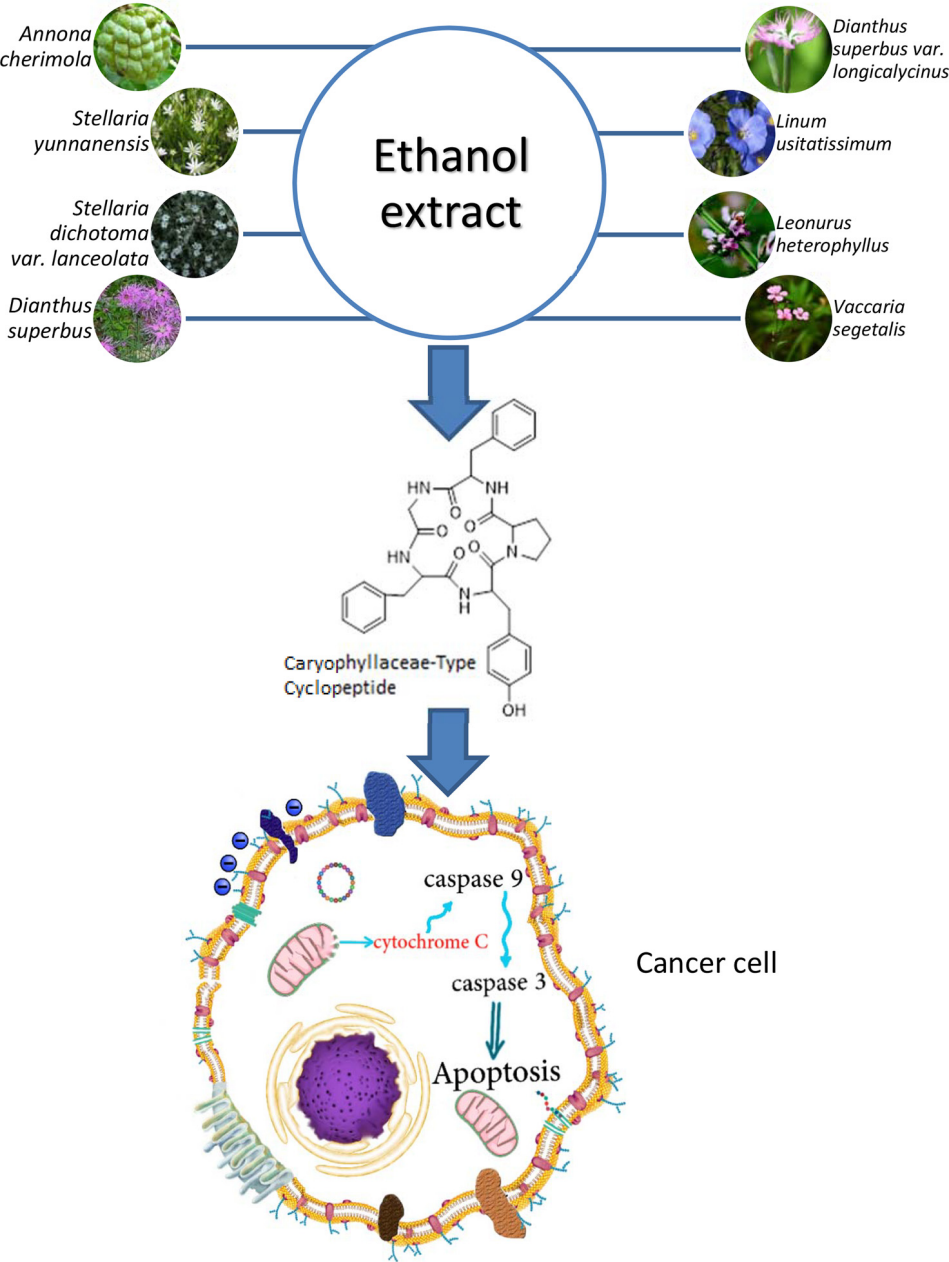
Caryophyllaceae-type cyclopeptides, isolated from the ethanol extract of various higher plants of Caryophyllaceae type and their cancer cell cytotoxicity.

Caryophyllaceae-type cyclopeptides, as natural peptides isolated from the ethanol extract of various higher plants of Caryophyllaceae type (**Figure 10**), have shown anticancer activity against several cancerous cell lines. The common feature in these peptides, apart from the cyclic structure, is the hydrophobic characteristic of their whole molecules due to the high amount of nonpolar amino acid residues present in their structures (≈80%). As can be roughly calculated from **Table 8**, proline, phenylalanine, and glycine, in order, make up the higher percentage of residue content in the cyclopeptide scaffolds compared with other residues. Polar amino acids make less contribution (around 20%) in the cyclic structures of peptides. Therefore, from the point of structure–activity relationship (SAR) studies, it may be estimated that these naturally occurring cyclopeptides insert their toxicity on cancer cells by a hydrophobic/hydrophilic characteristic balanced equal to a 4/1 ratio. This estimate is in accordance with a study that reports higher hydrophobic peptides infuse better into the cancer cells through the nonpolar part of the cell membrane, which results in cell disruption and necrosis (82, 83). Moreover, the presence of proline as well as glycine in the peptides is important for interaction with the cancer cell membrane (84). Phenylalanine residue can also increase the affinity of the peptides for interaction with the membrane of cancer cells (85). On the other hand, tyrosine residue as a polar amino acid may raise the toxicity of the peptides toward cancer cells (83, 86). In addition, the cyclic form of the peptides with fixed conformational structure and less occupying space can cause peptides to face less of a physical barrier to enter the cells (87). However, modification of these cyclic peptides by chemical means, either in a hydrophobic direction (e.g., acylation) or in a hydrophilic way (e.g., phosphorylation to impose negative charge or replacing/adding lysine or arginine within the cyclic peptide structure to add positive charge), is needed (88). By such favorable modification, these cyclic peptides can meet most all the requirements of SAR data needed to make them good candidates for cancer treatment in the clinic. On the other hand, conjugation of these peptides through tyrosine residue with an anticancer drug may even increase their physicochemical properties. These peptides considered cell-penetrating/targeting peptides can improve specificity and reduce side effects of the present anticancer drugs.

**Table 8:** Caryophyllaceae-type cyclopeptides as cytotoxic agents, comparing the properties of amino acid (AA) content in their sequences. Each (+) stands for one AA. AAs with colored names indicate their importance in cytotoxic activity of the cyclopeptides.

Peptide name	Sequence	AA, acidic	AA, basic	AA, polar	AA, nonpolar
Segetalin E	Cyclo(-Gly-Tyr-Val-Pro-Leu-Trp-Pro-)			++	+++++
Yunnanin A	Cyclo(-Gly-Tyr-Gly-Gly-Pro-Phe-Pro)			++++	+++
Yunnanin B	Cyclo(-Gly-Ser-δ-HO Ile-Phe-Phe-Ala)			+++	+++
Yunnanin C	Cyclo(-Gly-Ile-Gly-Phe-Tyr-Ser-Pro)			++++	+++
Yunnanin D	Cyclo(-Gly-Ile-Ser-Phe-Arg-Phe-Pro)		+	++	++++
Dichotomin A	Cyclo(-Gly-Thr-Phe-Leu-Tyr-Val)			+++	+++
Dichotomin B	Cyclo(-Gly-Thr-Phe-Leu-Tyr-Thr)			++++	++
Dichotomin C	Cyclo(-Gly-Thr-Phe-Leu-Tyr-Ala)			+++	+++
Dichotomin E	Cyclo(-Gly-Tyr-Ala-Phe-Ala)			++	+++
Dichotomin H	Cyclo(Ala-Pro-Thr-Phe-Tyr-Pro-Leu-Ile)			++	++++++
Dichotomin I	Cyclo(-Val-Pro-Thr-Phe-Tyr-Pro-Leu-Ile)			++	++++++
Cycloleonuripeptide B	Cyclo(-Gly-Pro-Pro-Pro-Tyr-Pro-Pro-Met(O)-Ile)			+++	++++++
Cycloleonuripeptide C	Cyclo(-Gly-Pro-Pro-Pro-Tyr-Pro-Pro-Met(O)-Ile)			+++	++++++
Cherimolacyclopeptide A	Cyclo(-Pro-Gln-Thr-Gly-Met-Leu-Pro-Ile)			**+++**	**+++++**
Cherimolacyclopeptide B	Cyclo(-Pro-Gln-Thr-Gly-OMt-Leu-Pro-Ile)			**++++**	**++++**
Dianthin A	cyclo-(Ala-Tyr-Asn-Phe-Gly-Leu)			**++**	**++++**
Dianthin C	cyclo-(Gly-Pro-Phe-Tyr-Val-Ile)			**++**	**++++**
Dianthin D	cyclo-(Gly-Ser-Leu-Pro-Pro-Ile-Phe)			**++**	**+++++**
Dianthin E	cyclo-(Gly-Pro-Ile-Ser-Phe-Val)			**++**	**++++**
Dianthin F	cyclo-(Gly-Pro-Phe-Val-Phe)			**+**	**++++**
Dianthin H	cyclo-(Gly-Pro-Val-Thr-Ile-Phe)			**++**	**++++**
Longicalycinin A	cyclo-(Gly-Phe-Tyr-Pro-Phe).			**++**	**+++**
Cyclolinopeptide A	Cyclo(-Pro-Pro-Phe-Phe-Leu-Ile-Ile-Leu-Val)				**+++++++++**
Cyclolinopeptide B	Cyclo(-Pro-Pro-Phe-Phe-Val-Ile-Met-Leu-Ile)				**+++++++++**
Cyclolinopeptide E	Cyclo(-Pro-Leu-Phe-Ile-OMet-Leu-Val-Phe)			**+**	**+++++++**

Because medicinal plants containing cyclopeptides, including caryophyllaceae-type cyclopeptides, have been used traditionally for a long time, it is inferred that these cyclopeptides should not be toxic to healthy cells/tissues of human body, so this is another reason for considering these cyclopeptides as safer compounds for cancer therapy compared with the conventional chemotherapy agents. However, modified caryophyllaceae-type cyclopeptides, like any other newly introduced agent as drugs, must pass all the requirements necessary to ensure that these cyclopeptides are safe as well as biologically active enough to add to the treatment schedules of cancer therapy.

## Author Contributions

A part of this manuscript concerning Longicalycinin A synthesis and its analogues was experimentally involved by MG, AB, and BM. All authors contributed to the article and approved the submitted version.

## Conflict of Interest

The authors declare that the research was conducted in the absence of any commercial or financial relationships that could be construed as a potential conflict of interest.
